# *Perilla frutescens* seeds enhance lamb immunity and antioxidant capacity via the microbiota-gut-liver-muscle axis

**DOI:** 10.1186/s40104-025-01317-3

**Published:** 2026-01-04

**Authors:** Yue Yu, Boyan Zhang, Xianzhe Jiang, Yimeng Cui, Yuqing Shang, Yanqin Jin, Tiancheng Sun, Ziwei Yuan, Zihan Zhang, Hailing Luo, Sokratis Stergiadis, Bing Wang

**Affiliations:** 1 State Key Laboratory of Animal Nutrition and Feeding, College of Animal Science and Technology, China Agricultural University, Beijing, 100193 People’s Republic of China; 2 Sanya Institute of China Agricultural University, Sanya, 57200 China; 3 School of Agriculture, Policy and Development, Department of Animal Sciences, University of Reading, Reading, RG6 6EU UK

**Keywords:** Gut microbiota, Immunomodulatory, Lamb, Liver, Metabolome

## Abstract

**Background:**

*Perilla frutescens* seeds (PFS) are gaining recognition as a natural alternative to antibiotics in livestock, supporting sustainable farming and animal health. However, the underlying molecular mechanisms through which PFS influence host immune function and antioxidant capacity, especially via the gut-liver-muscle axis, remain largely unknown. This study employed an integrative multi-omics approach to elucidate how PFS supplementation modulates the microbiota-gut-liver-muscle axis and enhances immune and antioxidant functions in lambs.

**Results:**

PFS supplementation markedly improved immune and antioxidant profiles, demonstrated by elevated serum levels of IL-10, IgM, IgG, GSH-PX, and SOD, and reductions in IL-1β, TNF-α, and MDA. Microbial analysis revealed elevated abundances of ruminal and intestinal taxa commonly associated with gut homeostasis and metabolic health (*Christensenellaceae_R-7_group*) and reduced levels of species with pathogenic or pro-inflammatory potential (*Bacillus cereus* and *Clostridioides*) in the ileum. Transcriptomic and metabolomic profiling of liver tissue indicated modulation of key inflammatory and bile acid signaling pathways, including the downregulation of *TLR4*, *NLRP3*, *ATF3*, *CYP2J2*, and *LXR-α*. PFS also increased hepatic concentrations of anti-inflammatory metabolites such as chlorquinaldol and indole-3-carboxaldehyde, while reducing levels of LysoPC(20:4) and phosphatidic acid. Correlation and mediation analyses revealed strong interconnections among gut microbiota, hepatic gene expression, lipid metabolites in liver and muscle, and systemic immune-antioxidant markers.

**Conclusion:**

These findings highlight the microbiota-gut-liver-muscle axis as a central mechanism through which PFS enhances immune function and antioxidant capacity in lambs. PFS supplementation represents a promising nutritional strategy to improve healthy lamb production, supporting the development of antibiotic-free and sustainable livestock systems.

**Supplementary Information:**

The online version contains supplementary material available at 10.1186/s40104-025-01317-3.

## Background

Ensuring animal health is central to sustainable livestock production and aligns with the One Health approach, which recognises the interconnectedness of animal, human, and environmental health [1]. Among the mechanisms that support animal well-being, the gut-liver axis plays a pivotal role in coordinating immune function, maintaining metabolic balance, and safeguarding the quality and safety of animal-derived foods [2, 3]. This bidirectional communication network—mediated through the bile ducts, portal vein, and systemic circulation—enables gut-derived metabolites to influence liver function and systemic immunity [3]. Microbial metabolites such as bile acids, indoles, and short-chain fatty acids not only fortify the host against pathogens but also regulate hepatic metabolism and immune responses [4, 5].

Dietary strategies offer an effective means to modulate this axis and improve animal health outcomes. Plant-based foods, rich in bioactive compounds such as flavonoids and polyphenols, have shown particular promise in promoting liver health and supporting gut-liver crosstalk [2]. *Perilla frutescens*, a member of the Labiatae family, is widely used in East Asian cuisine and traditional medicine [6]. *Perilla frutescens* seeds (PFS) are rich in oil (30%–45%), particularly α-linolenic acid (50%–62%), and also contain bioactive secondary metabolites such as flavonoids (e.g., luteolin), phenylpropanoids, and terpenoids [7, 8]. α-Linolenic acid (ALA) has well-documented anti-inflammatory, neuroprotective, and cardioprotective effects and plays a key role in preventing metabolic disorders such as atherosclerosis and fatty liver disease [9]. In humans, omega-3 polyunsaturated fatty acids (n-3 PUFAs) improve mitochondrial function, modulate bile acid metabolism, and promote liver health [10, 11], with studies showing that dietary n-3 PUFA supplementation can mitigate non-alcoholic fatty liver disease through reductions in hepatic lipid accumulation and injury [12]. These compounds exert immunomodulatory and antioxidant effects by enhancing immune cell activity, modulating lymphocyte ratios, and influencing macrophage polarisation [13]. For example, luteolin has been shown to alleviate oxidative stress and protect intestinal integrity [14], while dietary polyphenols in general can modulate host immunity by shaping the gut microbiota [15].

Our previous research demonstrated that PFS supplementation improved growth performance, carcass yield, and meat quality in lambs [8]. Given the central role of this axis in mediating systemic metabolic and immunological responses, we hypothesise that PFS enhances immune function and antioxidant capacity in lambs via gut-liver-muscle interactions. However, the mechanisms underlying these benefits—particularly how PFS modulates immune function and whether this is linked to meat quality through the gut-liver-muscle axis—remain largely unexplored. The present study aims to elucidate the molecular mechanisms and regulatory pathways by which PFS influences immune responses and antioxidant capacity in lambs. Through integrative multi-omics analyses, we seek to provide new insights into how dietary PFS contributes to animal health and supports sustainable and health-focused lamb production.

## Materials and methods

### Animal experimental design and sampling

Thirty male Tan-sheep (approximately 6 months old, body weight 25.1 ± 1.23 kg), which were about to enter the fattening period, were selected from a farm in the Ningxia Hui Autonomous Region, China. The animals were kept in a designated area of the original farm, separate from other unselected sheep. The Tan lambs were randomly assigned to three groups, with 15 animals per group. Each group was further divided into three blocks (pens), with five lambs per block. Using the RAND function in Excel, they were randomly assigned to two groups (*n* = 15 each): 1) a low-concentrate control diet (LC; 45:55 forage:concentrate ratio, dry matter (DM) basis, Supplementary Table S1); and 2) LC diet supplemented with additional 3% PFS (PFS; 3% DM basis). PFS used in this study contains mainly α-linolenic acid (57.8% of the total fatty acids) and a variety of bioactive compounds such as luteolin, 7-hydroxycoumarin, and emodin. Animals were fed the experimental diets for 12 weeks, including a 2-week adaptation period followed by a 10-week measurement period. The animal growth and feed intake results have been published in our previous study [8]. Animals were slaughtered at the end of the experiment, while 10 mL of blood was collected from the jugular vein into non-anticoagulant tubes. The tubes were centrifuged at 3,000 × *g* for 15 min at room temperature, and the serum was separated and stored in three tubes at −80 °C for further analysis of biochemical parameters. Rumen fluid, ileum contents, liver tissue, and *longissimus lumborum* muscle samples were also collected, immediately frozen in liquid nitrogen, and stored at −80 °C for subsequent determinations.

### Serum biochemical and rumen fermentation characteristics analysis

Serum levels of glucose (GLU), triglyceride (TG) were measured using Shanghai Kehua Bioengineering Institute’s commercial kits (Shanghai, China), and the levels of total superoxide dismutase (SOD), malondialdehyde (MDA), glutathione peroxidase (GSH-Px), immunoglobulin A (IgA), immunoglobulin G (IgG), immunoglobulin M (IgM), interleukin-1β (IL-1β), interleukin-10 (IL-10), tumor necrosis factor-alpha (TNF-α), and transforming growth factor-β (TGF-β) were measured using Nanjing Jiancheng Bioengineering Institute’s commercial kits (Nanjing, China) according to the manufacturer’s instructions. Rumen fluid pH was measured immediately using a calibrated Testo 205 pH meter (Testo GmbH, Germany) with automatic temperature compensation. Volatile fatty acids (VFAs) were analyzed via gas chromatography (Trace 1300; Thermo Fisher Scientific, Shanghai, China), and rumen ammonia-nitrogen (NH_3_-N) was determined using the phenol-sodium hypochlorite colorimetry method [16].

### 16S rRNA sequencing

Microbial DNA from rumen and ileum contents was extracted using HiPure Soil DNA Kits (Magen, Guangzhou, China) following the manufacturer’s instructions. The V3–V4 region of the 16S rRNA gene was amplified by PCR with the following primers: 341 F (5'-CCTACGGGNGGCWGCAG-3') and 806R (5'-GGACTACHVGGGTATCTAAT-3'). PCR conditions were: 94 °C for 2 min, followed by 30 cycles of 98 °C for 10 s, 62 °C for 30 s, and 68 °C for 30 s, with a final extension at 68 °C for 5 min. Reactions were performed in triplicate, with each 50 μL mixture containing 5 μL of 10 × KOD buffer, 5 μL of 2 mmol/L dNTPs, 3 μL of 25 mmol/L MgSO_4_, 1.5 μL of each primer (10 μmol/L), 1 μL of KOD polymerase, and 100 ng of template DNA. Amplicons were extracted from 2% agarose gels, purified using the AxyPrep DNA Gel Extraction Kit (Axygen Biosciences, Union City, CA, USA), and quantified using the ABI StepOnePlus Real-Time PCR System (Life Technologies, Foster City, USA). Purified amplicons were pooled in equimolar amounts and sequenced using paired-end sequencing (2 × 250) on an Illumina platform following standard protocols. Raw reads were filtered using FASTP (version 0.18.0) and deposited in the NCBI Sequence Read Archive (SRA) database. Paired-end reads were merged into original tags using FLASH (version 1.2.11), and noise sequences were removed to obtain high-quality clean tags. Filtered sequences were analyzed with the DADA2 pipeline to generate amplicon sequence variants (ASVs), and taxonomic assignments were conducted using the Naive Bayes pretrained SILVA database (version 138). Bioinformatics analysis was performed using QIIME2 (http://qiime.org).

### Liver transcriptomics analysis

Total RNA was extracted from liver tissue using the TRIzol reagent (Invitrogen, Carlsbad, CA, USA) following the manufacturer’s instructions. The RNA quality was assessed using an Agilent 2100 Bioanalyzer (Agilent Technologies, Palo Alto, CA, USA) and further verified through RNase-free agarose gel electrophoresis. Eukaryotic mRNA was enriched using Oligo(dT) beads, fragmented with a fragmentation buffer, and reverse-transcribed into cDNA using the NEBNext Ultra RNA Library Prep Kit for Illumina (New England Biolabs, Ipswich, MA, USA). The double-stranded cDNA fragments were purified, end-repaired, and an A base was added before ligating Illumina sequencing adapters. The ligation products were purified using AMPure XP Beads and amplified by PCR. The cDNA libraries were sequenced on the Illumina NovaSeq 6000 platform by Gene Denovo Biotechnology Co., Ltd. (Guangzhou, China).

### Liver metabolomics analysis

Liver tissue samples (25 ± 1 mg) were homogenized with beads in 500 μL of extraction solution (methanol:acetonitrile:water, 2:2:1, vol/vol) containing deuterated internal standards. The mixture was vortexed for 30 s, incubated at −40 °C for 1 h to precipitate proteins, and then centrifuged at 13,800 × *g* at 4 °C for 15 min. The supernatant was transferred to a clean vial for analysis. A quality control (QC) sample was prepared by pooling equal aliquots of the supernatants from all samples. Polar metabolites were analyzed using LC–MS/MS on a UHPLC system (Vanquish, Thermo Fisher Scientific) coupled with an Orbitrap Exploris 120 mass spectrometer (Thermo) and equipped with a Waters Acquity UPLC BEH Amide column (2.1 mm × 50 mm, 1.7 μm). The mobile phase consisted of 25 mmol/L ammonium acetate and 25 mmol/L ammonium hydroxide in water (pH 9.75) (A), and acetonitrile (B). The injection volume was set to 2 μL, and the auto-sampler was maintained at 4 °C. The Orbitrap Exploris 120 mass spectrometer, controlled by Xcalibur software, operated in information-dependent acquisition (IDA) mode to acquire MS/MS spectra. The ESI source conditions were as follows: sheath gas flow rate, 50 Arb; auxiliary gas flow rate, 15 Arb; capillary temperature, 320 °C; full MS resolution, 60,000; MS/MS resolution, 15,000; collision energy, SNCE 20/30/40; and spray voltage, 3.8 kV (positive mode) or −3.4 kV (negative mode). Raw data were converted to mzXML format using ProteoWizard and processed with an in-house R-based program, which employed XCMS for peak detection, extraction, alignment, and integration. Metabolite identification was performed using the R package and BiotreeDB (V3.0).

### Statistical, correlation, and mediation analysis

Spearman’s correlation analysis was conducted using the OmicShare platform (https://www.omicshare.com) to identify relationships between differential microbes, serum biochemical parameters, muscle metabolites that have been published previously [8], and rumen fermentation parameters (*P* < 0.01, *r* > |0.6|). Sankey diagrams were generated to visualize these relationships which was plotted by an online platform (https://www.bioinformatics.com.cn; last accessed on 10 Oct 2024). Mediation analysis was performed to assess the mediating effect of variables on the relationship between treatments and outcomes [17, 18]. The multi-omics data were standardized using Z-score normalization prior to modelling. This analysis was conducted using the R package “mediation” with the following parameters: bootstrapping enabled (boot = “TRUE”), confidence intervals set to percentile type (boot.ci.type = “perc”), a 95% confidence level (conf.level = 0.95), and 1,000 simulations (sims = 1,000). A sensitivity analysis was performed using the “medsens” R package to evaluate the robustness of the mediation effect and check for potential violations of the sequential ignorability assumption. The mediation results were reported in accordance with the AGReMA (Guideline for Reporting Mediation Analyses) statement [19]. Regularized generalized canonical correlation analysis (RGCCA) was also used for integrative multi-omics correlation analysis.

Serum biochemical and rumen fermentation parameters were analyzed using linear mixed models in IBM SPSS Statistics (version 26.0), with statistical plots generated in GraphPad Prism (version 9.0). Microbial sequencing data from the LC and PFS groups were compared using the Wilcoxon rank-sum test to identify differences between the rumen and ileum microbiomes (*P* < 0.05). Alpha diversity indices (ACE, Shannon, Chao1, and Simpson) were also compared using the Wilcoxon rank-sum test. Beta diversity was assessed using Bray–Curtis dissimilarity and weighted UniFrac distance and visualized through Principal Coordinates Analysis (PCoA). Comparisons of bacterial relative abundance between PFS and LC groups were conducted using the Wilcoxon rank test after using centered log-ratio (clr) transformation. Linear discriminant analysis effect size (LEfSe) was used to determine the differential abundance of bacterial taxa between samples (LDA score > 2.8). Analysis of compositions of microbiomes with bias correction (ANCOM-BC) were performed to identify critical microbial taxa (*P* < 0.05).

Differential gene expression analysis was performed using DESeq2 software. Genes with a *P*-value below 0.05 and |log_2_fold change (FC)| ≥ log_2_(1.5) were considered differentially expressed genes (DEGs). All DEGs were subjected to Kyoto Encyclopedia of Genes and Genomes (KEGG) pathway analysis (https://www.genome.jp/kegg/) to predict biological functions. Gene set enrichment analysis (GSEA) was used to identify significant differences in KEGG pathways between the two groups, with enrichment scores greater than 1 and a false discovery rate (FDR) below 0.05 considered significant. Weighted gene co-expression network analysis (WGCNA) was conducted on muscular differential metabolites using the cloud platform (https://cloud.oebiotech.com/) to identify module information.

Supervised orthogonal projections to latent structures-discriminant analysis (OPLS-DA) was used to visualize group separation and identify significantly altered metabolites. The variable importance in projection (VIP) score from the first principal component in OPLS-DA summarized each variable’s contribution to the model. Metabolites with VIP > 1 and *P* < 0.05 (Student’s *t*-test) were considered significantly altered. Additionally, pathway enrichment analysis was performed using the KEGG (http://www.genome.jp/kegg/) and MetaboAnalyst (http://www.metaboanalyst.ca/) databases. Metabolite traceability analysis was used by MetOrigin (https://metorigin.met-bioinformatics.cn/) for differential metabolites.

## Results

### Changes in immune and antioxidant activities in serum

**Fig. 1:**
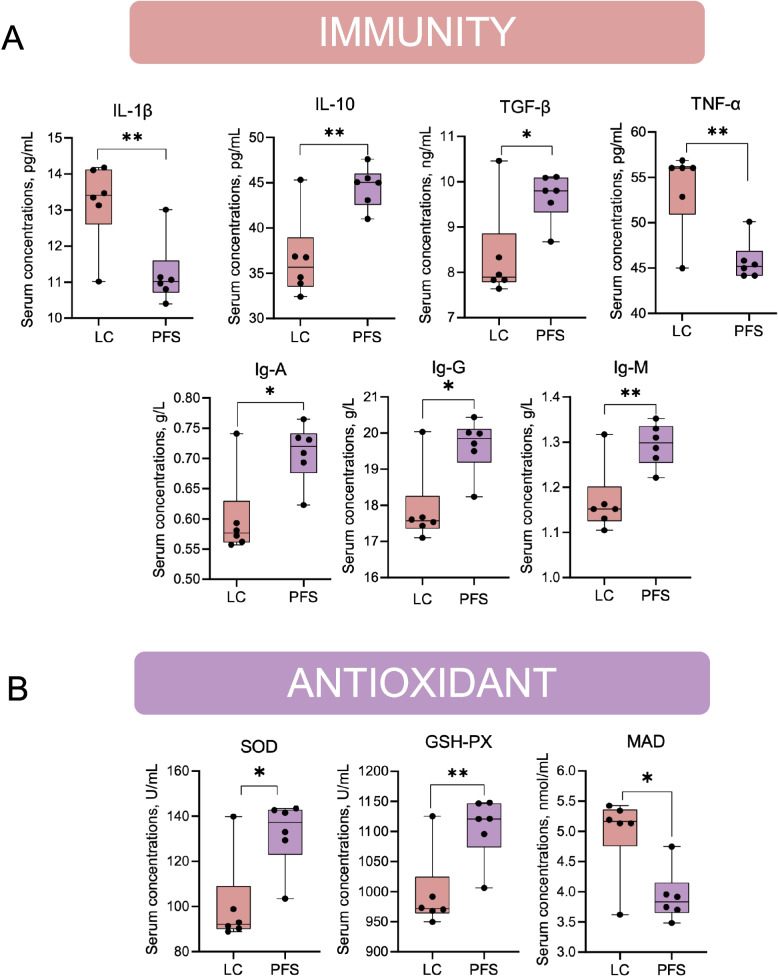
Effects of PFS supplementation on serum immune and antioxidant markers. **A** Serum concentrations of immune markers IL-1β, IL-10, TNF-α, TGF-β, IgG, IgM, and IgA. **B** Antioxidant capacity indicated by serum MDA, GSH-PX, and SOD levels. ^*^P < 0.05, ^**^P < 0.01

Compared to the LC group, the PFS group exhibited higher serum concentrations of IL-10, IgA, IgM, IgG, and TGF-β, and lower levels of IL-1β and TNF-α (*P* < 0.05, Fig. 1A). For the antioxidant index, the PFS increased the serum concentrations of GSH-Px and SOD, and decreased MDA content compared to the LC group (*P* < 0.05, Fig. 1B).

### Rumen fermentation characteristics

**Fig. 2:**
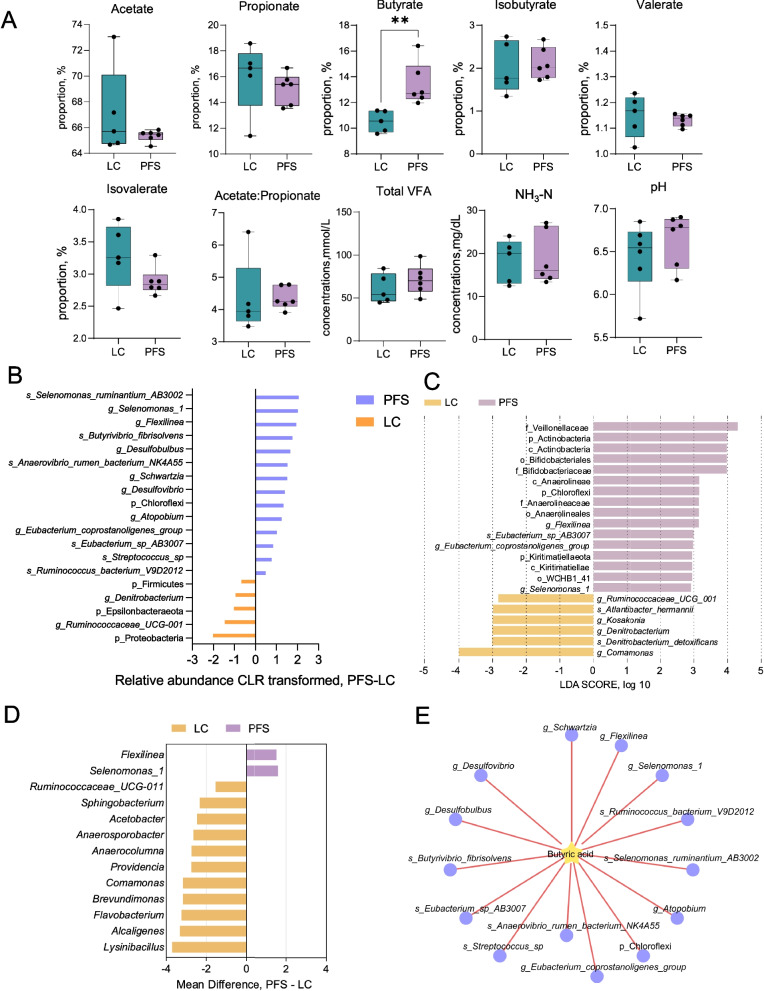
Rumen fermentation parameters and microbial community alterations induced by PFS supplementation. **A** Rumen pH, ammonia-N (NH_3_-N), and volatile fatty acid (VFA) concentrations and proportions. **B** Differentially abundant rumen bacteria identified by Wilcoxon rank-sum test. **C** LEfSe analysis highlighting differential rumen bacteria. **D** Differential rumen bacterial genera (ANCOM-BC, *P* < 0.05). **E** Spearman correlation analysis linking rumen butyrate proportion with bacterial taxa. ^**^P < 0.01

The PFS group demonstrated a significant increase in butyrate concentration compared to the LC group (*P* < 0.01), while no significant differences were observed in other rumen fermentation parameters (Fig. 2A).

### Rumen and ileum microbial structure and function

For the rumen microbiota analysis, the Sob index dilution curves plateaued, indicating sufficient sequencing coverage. The alpha and beta-diversity analysis showed no apparent distinction and differences between the LC and PFS groups for rumen (Supplementary Fig. S1A). At the phylum level, Firmicutes, Bacteroidetes, Proteobacteria, Euryarchaeota, and Patescibacteria were dominant in both groups, collectively accounting for over 90% of the rumen microbiota (Supplementary Fig. S1B). At the genus level, the most abundant taxa included *Lysinibacillus*, *Prevotella*_1, *Rikenellaceae*_RC9-gut-group, and *Alcaligenes* (Supplementary Fig. S1C). The PFS group exhibited significantly higher relative abundances of *Selenomonas_ruminantium*_AB3002, *Flexilinea*, *Selenomonas*_1, *Schwartzia*, *Desulfovibrio*, *Desulfobulbus*, *Butyrivibrio*_*fibrisolvens*, *Streptococcus*, and *Eubacterium_coprostanoligenes*_group, while lower abundances were observed for *Ruminococcaceae*_UCG-001 and *Denitrobacterium* (*P* < 0.05; Fig. 2B). LEfSe analysis further confirmed enrichment of Actinobacteria, Chloroflexi, and Kiritimatiellaeota at the phylum level, and *Flexilinea*, *Eubacterium*_*coprostanoligenes*_group, and *Selenomonas*_1 at the genus level. Conversely, *Ruminococcaceae*_UCG-001, *Kosakonia*, *Denitrobacterium*, and *Comamonas* were significantly lower in the PFS group. At the species level, *Eubacterium*_sp_AB3007 was significantly increased, whereas *Atlantibacter hermannii* and *Denitrobacterium detoxificans* were reduced in the PFS group (Fig. 2C). ANCOM-BC analysis indicated significantly higher absolute abundances of *Selenomonas*_1 and *Flexilinea* in the PFS group, alongside significantly lower absolute abundances of *Lysinibacillus*, *Alcaligenes*, *Flavobacterium*, *Brevundimonas*, *Comamonas*, *Providencia*, *Anaerocolumna*, *Anaerosporobacter*, *Acetobacter*, *Sphingobacterium*, and *Ruminococcaceae*_UCG-011 (Fig. 2D). Additionally, rumen butyrate concentrations exhibited significant positive correlations with multiple bacterial genera, including *Eubacterium_coprostanoligenes*_group, *Flexilinea*, *Butyrivibrio fibrisolvens*, *Selenomonas*_1, *Selenomonas_ruminantium*_AB3002, *Desulfobulbus*, and *Ruminococcus*_UCG-001 (Fig. 2E).

**Fig. 3:**
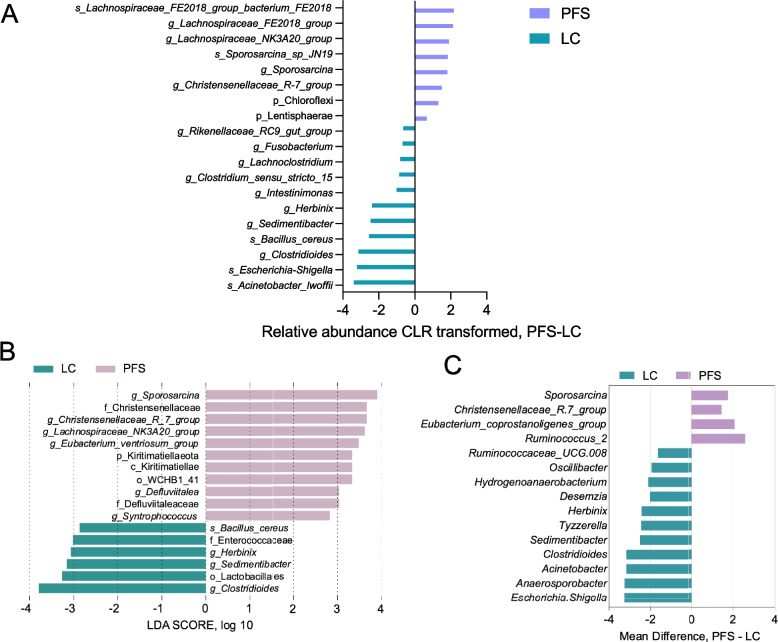
Ileal bacterial community composition after PFS supplementation. **A** Differentially abundant ileal bacteria identified by Wilcoxon rank-sum test. **B** LEfSe analysis highlighting differential ileal bacteria. **C** Differential ileal bacterial genera (ANCOM-BC, *P* < 0.05)

For the ileum microbiota analysis, the Sob index dilution curves plateaued, indicating sufficient sequencing coverage. The alpha and beta-diversity analysis showed no apparent distinction and differences between the LC and PFS groups (Fig. S2A). the dominant phyla (relative abundance > 1%) were Firmicutes, Proteobacteria, and Patescibacteria (Fig. S2B). At the genus level, after excluding unclassified genera, the microbiota was dominated by *Lysinibacillus*, *Acinetobacter*, *Anaerocolumna*, *Providencia*, *Anaerosporobacter*, *Bacillus*, *Tissierella*, and *Clostridium*_*sensu*_stricto_1 (Supplementary Fig. S2C). Compared to the LC group, the PFS group exhibited higher relative abundances of Lentisphaerae (phylum level), *Sporosarcina*, *Christensenellaceae*_R-7_group, *Sporosarcina*_sp_JN19, *Lachnospiraceae*_NK3A20_group, and *Lachnospiraceae*_FE2018_group at the genus or species level. In contrast, the relative abundances of *Herbinix*, *Sedimentibacter*, *Bacillus*_*cereus*, *Clostridioides*, and *Escherichia*_*Shigella* were reduced in the PFS group (*P* < 0.05, Fig. 3A). LEfSe analysis (LDA ≥ 2.8) identified greater relative abundances of *Kiritimatiellaeota*, *Sporosarcina*, *Christensenellaceae*_R-7_group, *Defluviitale*, *Lachnospiraceae*_NK3A20_group, *Eubacterium*_*ventriosum*_group, and *Syntrophococcus* in the PFS group, while *Herbinix*, *Sedimentibacter*, *Clostridioides*, and *Bacillus*_*cereus* were lower in the PFS group (Fig. 3B). ANCOM-BC analysis (*P* < 0.05) revealed that the PFS group had lower absolute abundances of *Escherichia*-*Shigella*, *Anaerosporobacter*, *Acinetobacter*, *Clostridioides*, *Sedimentibacter*, *Tyzzerella*, *Herbinix*, *Desemzia*, *Hydrogenoanaerobacterium*, *Oscillibacter*, and *Ruminococcaceae*_UCG-008, while *Ruminococcus*_2, *Eubacterium*_*coprostanoligenes*_group, *Christensenellaceae*_R-7_group, and *Sporosarcina* were higher compared to the LC group (Fig. 3C).

### Gene expression in liver tissue

**Fig. 4:**
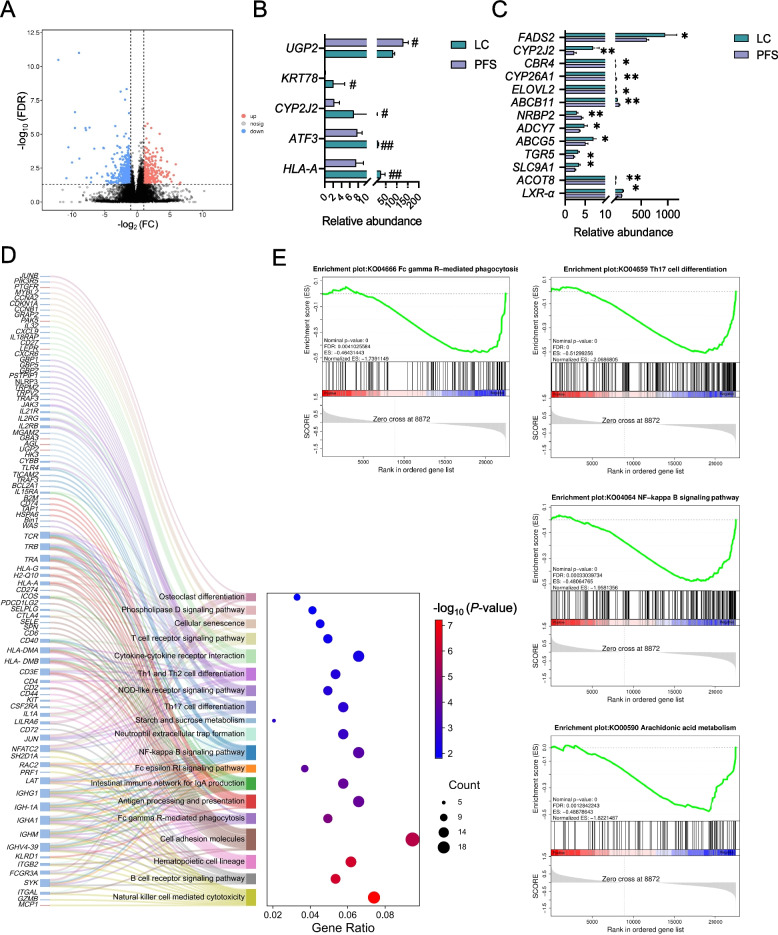
Liver transcriptomic responses to PFS supplementation. **A** Volcano plot showing differential gene expression between LC and PFS groups (*P* < 0.05 and |log_2_FC| ≥ log_2_(1.5)). **B** Differentially expressed genes identified with FDR correction (^#^FDR < 0.05, ^##^FDR < 0.01). **C** Differential genes specifically involved in lipid and bile acid metabolism. **D** Top 20 KEGG pathways enriched by differentially expressed genes. **E** Gene Set Enrichment Analysis (GSEA) highlighting significantly enriched pathways (FDR < 0.05, *P* < 0.01). ^*^P < 0.05, ^**^P < 0.01

A total of 481 DEGs were identified between the LC and PFS groups (|FC| ≥ log_2_1.5, *P* < 0.05), with 127 genes up-regulated and 354 down-regulated in the PFS group (Fig. 4A, Supplementary Table S2). And five of the DEGs had an FDR < 0.05, of which *HLA-A, ATF3, CYP2J2*, and *KRT78* were down-regulated in the PFS group and *UGP2* was up-regulated in the PFS group (Fig. 4B). Genes associated with bile acid metabolism and fatty acids metabolism, such as *CBR4*, *CYP26A1*, *ELOVL2*, *ABCB11* and *NRBP2*, were up-regulated, while *CYP2J2*, *ADCY7*, *ABCG5*, *TGR5*, *SLC9A1*, *ACOT8*, and *LXR*-α were down-regulated in the PFS group (Fig. 4C). Notably, immune-related genes such as *HLA-A*, *HLA-DMB*, *CD4*, *CD40*, *JAK3*, *TLR4*, *NLRP3*, and *FADS* were significantly down-regulated in the PFS group compared to the LC group. KEGG pathway enrichment analysis of the hepatic DEGs revealed that 13 out of the top 20 enriched pathways were immunologically relevant (Fig. 4D). These pathways included cytokine-cytokine receptor interaction, Th17 cell differentiation, Th1 and Th2 cell differentiation, phagosome, natural killer cell-mediated cytotoxicity, B cell receptor signaling, Fc gamma R-mediated phagocytosis, antigen processing and presentation, IgA production, Fc epsilon RI signaling, NF-kappa B signaling, and T cell receptor signaling pathways (Fig. 4D). Gene Set Enrichment Analysis (GSEA) of 22,488 genes revealed 328 enriched pathways, with 134 pathways up-regulated in the PFS group. Of these, 114 pathways were significant at an FDR < 0.25, and 75 gene sets were significantly enriched at a nominal *P*-value < 0.01. Notably, pathways such Fc gamma R-mediated phagocytosis, Th17 cell differentiation, NF-kappa B signaling, and arachidonic acid metabolism were significantly down-regulated in the PFS group (FDR < 0.05, *P* < 0.01) (Fig. 4E).

### Liver metabolome and metabolic pathways

**Fig. 5:**
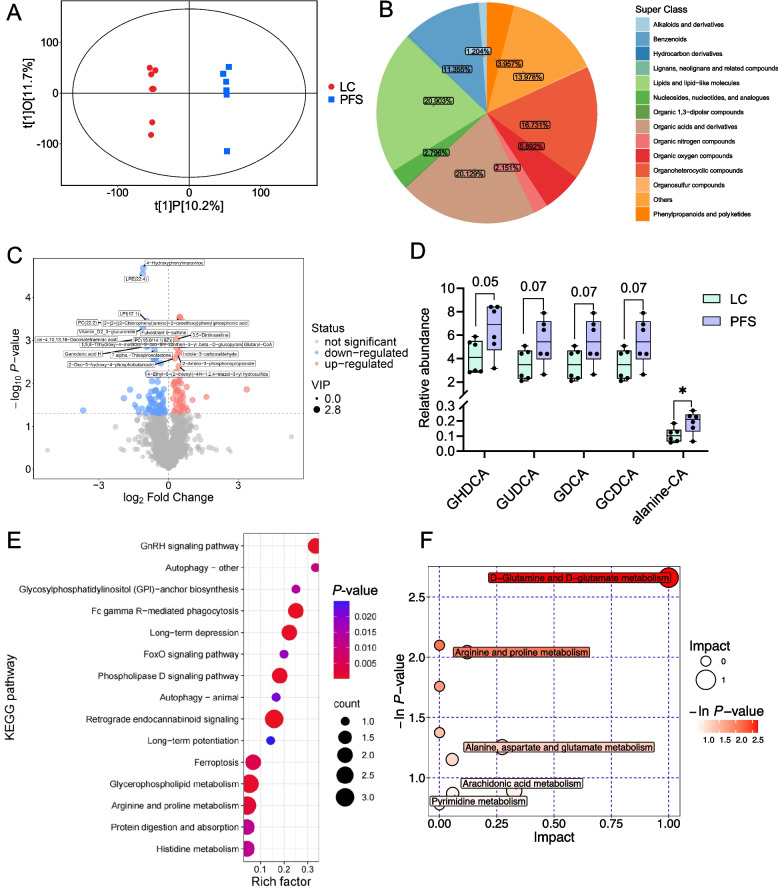
Hepatic metabolomic profiling following PFS supplementation. **A** Orthogonal Projections to Latent Structures Discriminant Analysis (OPLS-DA) score plot comparing liver metabolites between LC and PFS groups. **B** Classification of hepatic metabolites into functional categories. **C** Volcano plot highlighting significantly altered liver metabolites (VIP > 1, *P* < 0.05). **D** Changes in hepatic bile acid composition induced by PFS. **E** Top 15 enriched KEGG pathways based on metabolite alterations. **F** Metabolic pathway overview generated by MetaboAnalyst. ^*^P < 0.05

A total of 39,905 peaks were detected. After applying relative standard deviation de-noising, 2,920 metabolites remained, of which 2,325 were identified as secondary qualitative substances. OPLS-DA revealed a clear separation of compounds between the two groups (R^2^Y = 0.998, Q^2^ = 0.282, Fig. 5A). 2,325 metabolites were classified into various categories including lipids and lipid-like molecules (20.90%), organic acids and derivatives (20.13%), organic oxygen compounds (5.89%), phenylpropanoids and polyketides (3.96%), and others (Fig. 5B). A total of 126 different metabolites were identified between the two groups (VIP > 1, *P* < 0.05, Fig. 5C). Of these, 46 metabolites were up-regulated in the PFS group, including chlorquinaldol, swainsonine, and indole-3-carboxaldehyde. Conversely, 80 metabolites were down-regulated in the PFS group, including LPE(22:4), LPI(17:1), PC(22:2), vitamin D_2_ 3-glucuronide, *cis*-4,10,13,16-docosatetraenoic acid, fulvestrant 9-sulfone, PC(15:0/14:1(9Z)), ganoderic acid A, B, and H, geniposidic acid, apigetrin, syringin, nicotinamide N-oxide, and 2'-deoxyuridine (Supplementary Table S3). Specifically, the level of alanine-conjugated cholic acid (alanine-CA) was increased (*P* < 0.05) and 4 glycine-conjugated bile acids including glycohyodeoxycholic acid (GHDCA), glycoursodeoxycholic acid (GUDCA), glycodeoxycholic acid (GDCA), and glycochenodeoxycholic acid (GCDCA) tended to be higher in the PFS group (0.05 < *P* < 0.1) (Fig. 5D). KEGG enrichment analysis identified several pathways related to the differential metabolites, including retrograde endocannabinoid signaling, arginine and proline metabolism, glycerophospholipid metabolism, Fc gamma R-mediated phagocytosis, GnRH signaling, and phospholipase D signaling (Fig. 5E). The bubble plot further revealed pathways enrichment in D-glutamine and D-glutamate metabolism, arginine and proline metabolism, alanine, aspartate, and glutamate metabolism, arachidonic acid metabolism, and pyrimidine metabolism (Fig. 5F).

### Associations between gut microbiota, hepatic genes and metabolites, muscle metabolites, and serum immune and antioxidant index

WGCNA of muscle metabolites identified a ‘turquoise’ module comprising 37 core metabolites significantly associated with serum immune-antioxidant traits across both dietary groups (*P* < 0.05, Supplementary Fig. S3A). Notably, 18 of these ‘turquoise’ module core metabolites were lipids, primarily phosphatidylcholines (PCs) and lysophosphatidylcholines (LPCs), indicating their central role in mediating systemic effects of PFS supplementation (Supplementary Fig. S3B).

**Fig. 6:**
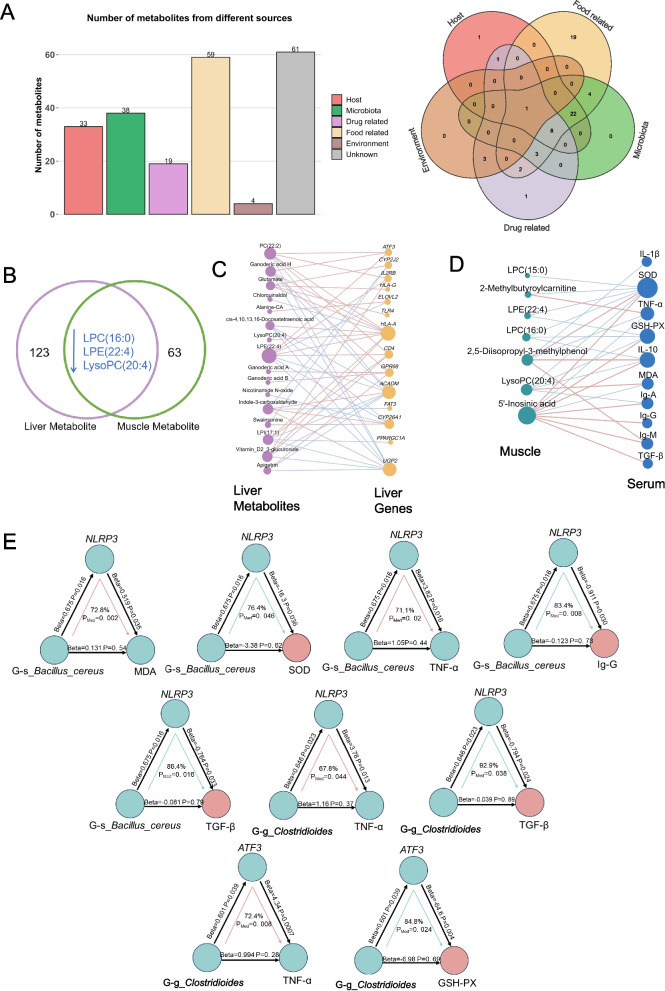
Multi-omics integration linking gut microbiota, hepatic metabolites, and host metabolism. **A** Source-based classification of differential hepatic metabolites (host-derived, microbiota-derived, drug-related, food-related, environmental, and others). **B** Comparative analysis between differential muscle and liver metabolites.** C** Correlation network of differential liver metabolites with liver genes. **D** Correlation network between muscle metabolites and serum immune-antioxidant indices (blue: negative correlation, pink: positive correlation). **E** Mediation analysis identifying hepatic genes mediating microbiota effects on host immunity and antioxidant capacity (cyan: negative mediation, red: positive mediation)

Among liver-derived metabolites, 126 were classified by origin into six categories: host, microbiota, food, drug, environmental, and unknown (Fig. 6A). Geniposidic acid, apigetrin, and syringin were food-derived, while chlorquinaldol was drug-related (Supplementary Table S4). Cross-tissue comparison identified three metabolites—LPC(16:0), LPE(22:4), and LysoPC(20:4)—downregulated in both liver and muscle following PFS supplementation (Fig. 6B). Hepatic LysoPC(20:4) was positively associated with hepatic *HLA-A* expression (Fig. 6C). 5′-Inosinic acid, elevated in muscle with PFS, showed strong positive associations with serum antioxidant and immune indices (SOD, IL-10, GSH-Px, TGF-β, IgM, IgA, IgG) and inverse correlations with MDA and TNF-α. In contrast, LysoPC(20:4) negatively correlated with SOD, GSH-Px, and IgA and positively with MDA (Fig. 6D).

Mediation analysis further delineated the mechanistic links between gut microbes, hepatic regulators, and host immune function and antioxidant capacity phenotype (Fig. 6E). Hepatic *NLRP3* mediated the positive association between ileal *Bacillus cereus* and serum MDA (72.8% mediation; *P* = 0.002) and serum TNF-α (71.1% mediation; *P* = 0.02). *NLRP3* mediated the negative association between ileal *Bacillus cereus* and serum SOD (76.4% mediation; *P* = 0.046), IgG (83.4% mediation; *P* = 0.008), and TGF-β (86.4% mediation; *P* = 0.016). *NLRP3* also mediated the positive and negative association between ileal *Clostridioides* and serum TNF-α (67.8% mediation; *P* = 0.044) and TGF-β (92.9% mediation; *P* = 0.038), respectively. *ATF3* mediated the positive and negative association between ileal *Clostridioides* and serum TNF-α (72.4% mediation; *P* = 0.008) and GSH-PX (84.8% mediation; *P* = 0.024), respectively.

The integrative multi-omics analysis revealed a clear shift in gut-liver cross-omics coordination between the LC and PFS groups using RGCCA (Supplementary Fig. S3C). In the LC-only mode, rumen microbiota exhibited a strong positive association with hepatic metabolites (*r* = 0.94). Hepatic transcriptomic activity was negatively correlated with liver metabolite levels (*r* = –0.99). In contrast, A positive correlation was found between liver transcripts and metabolites (*r* = 0.91). Negative feedback was found from liver metabolites to rumen bacteria (*r* = –0.92) and ileum bacteria (*r* = –0.91).

## Discussion

**Fig. 7:**
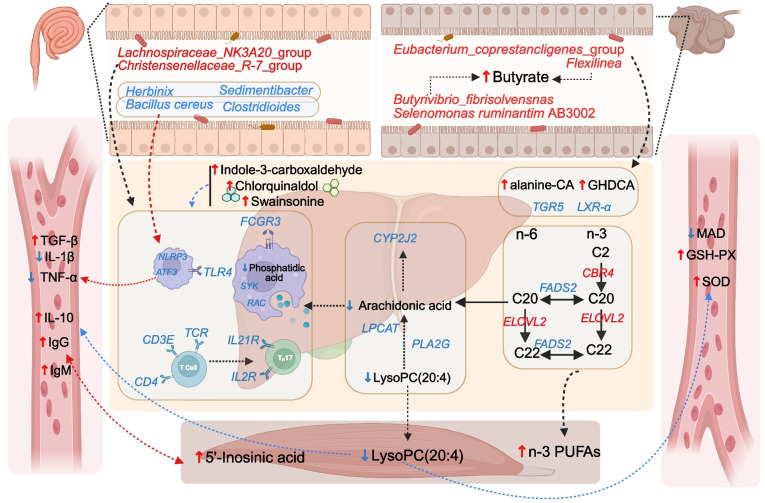
Integrated pathways modulated by PFS through the gut-liver-muscle axis. Schematic representation illustrating the integrated signalling and metabolic pathways influenced by *Perilla frutescens* seed (PFS) supplementation. PFS enhanced microbial butyrate production in the gut. In the liver, PFS promoted hepatic synthesis of PUFAs via upregulation of *ELOVL2* and *FADS2*, and modulated glycerophospholipid metabolism through suppression of *PLA2G* and *LPCAT*, resulting in reduced LysoPC(20:4) levels. Enhanced bile acid metabolism was characterised by increased GHDCA and CA, activating bile acid receptor TGR5 signalling. In immune-related pathways, PFS upregulated FcγR-mediated phagocytosis and T-cell receptor (TCR) signalling, while suppressing pro-inflammatory mediators (IL-1β, TNF-α) and enhancing anti-inflammatory cytokines (IL-10, TGF-β). The overall regulation of arachidonic acid metabolism and reinforcement of antioxidant defence collectively contribute to improved immune resilience and metabolic balance along the gut-liver-muscle axis. Red indicates upregulated elements, and blue indicates downregulated elements in response to PFS supplementation. CA: cholic acid; GHDCA: glycohyodeoxycholic acid; PUFAs: polyunsaturated fatty acids

Ensuring livestock health and improving the safety and quality of animal-derived foods are central objectives of the One Health framework, which integrates human, animal, and environmental health [20]. Our findings showed that dietary PFS supplementation significantly enhanced lamb immune and antioxidant status. Specifically, serum levels of IL-10, IgM, IgG, GSH-PX, and SOD were elevated, while IL-1β, TNF-α, and MDA were reduced*.* These physiological improvements, together with previously reported enhancements in meat quality [8], support the use of PFS as a potential feed additive or functional dietary component [21]. The gut-liver axis highlights the interconnectedness between gut microbiota, liver metabolism, and overall animal health and immunity [22]. Using multi-omics analysis across the gut-liver-muscle axis, we established an underlying metabolic framework illustrating how PFS improves lamb health (Fig. 7).

The regulation of gastrointestinal fermentation and microbial balance significantly affects ruminant health and productivity [23]. Our results revealed that PFS supplementation enriched the abundance of butyrate-producing microbes, such as *Eubacterium_coprestancligenes*_group [24, 25]. Butyrate, a key energy source, has immunomodulatory properties that enhance growth performance, nutrient digestion, and immune function [26]. Additionally, beneficial gut bacteria such as *Lachnospiraceae*_NK3A20_group and *Christensenellaceae*_R-7_group were significantly increased by PFS, supporting intestinal barrier integrity and reducing inflammation [27]. Previous research highlighted a positive correlation between *Lachnospiraceae*_NK3A20_group and butyrate production in sheep under inflammatory stress [28], while lower proportions of *Christensenellaceae* are indicative of metabolic disturbances in obesity [29]. Moreover, the enrichment of bile acid-metabolizing microbes (*Eubacterium* and *Christensenellaceae*_R-7_group) further confirms the role of PFS in bile acid transformation. Changes in amino acid-conjugated bile acids and downregulation of *TGR5* and *LXR*-α genes indicate the regulation of bile acid signaling pathways linked to immune modulation [30–33].

Using RGCCA to integrate hepatic transcriptomic and metabolomic blocks, we observed block-wise component correlations that diverged by diet. In the LC group, the strongly negative transcriptome–metabolome correlation is consistent with a decoupling between gene expression and metabolite turnover—an organisational pattern frequently associated with metabolic stress [34]. By contrast, the PFS group exhibited a positive cross-block correlation, indicating greater coupling between transcriptional responses and metabolite dynamics. Given that perilla oil is rich in α-linolenic acid, which is known to modulate hepatic transcriptional programmes, reduce lipid/oxidative stress, and improve metabolic homeostasis [35], these results support the interpretation that dietary PFS promotes an integrated metabolic–transcriptional feedback in the liver, in clear contrast to the decoupled, stress-aligned state observed under LC conditions.

Integrated transcriptomic and metabolomic analysis revealed that PFS modulated key hepatic pathways linked to inflammation and immune regulation. In particular, genes involved in Fc gamma receptor (FcγR)-mediated phagocytosis, such as IGH and FCGR3, were significantly downregulated. These gene changes correlated with reductions in phosphatidic acid and arachidonic acid, prominent inflammatory mediators [36]. Activation of FcγRs in the absence of infection typically promotes inflammatory responses via cytokines like IL-1β and TNF-α [37, 38]. Phosphatidic acid functions as a critical signaling molecule linking nutrient sensing to inflammatory pathways through its activation of the mTORC1 complex, which subsequently enhances NF-κB signaling [39]. Additionally, spleen tyrosine kinase (SYK) plays a central role in inflammation via FcγR-dependent signaling pathways [40]. Upon activation, SYK triggers phosphorylation events involving PI3K/Akt signaling, ultimately facilitating NF-κB activation [41]. Th17 cells are known to play a central role in hepatic inflammation and injury [42]. In our study, PFS supplementation reduced the expression of IL-21R, IL-2R, and components of the NF-κB signaling pathway. Since these receptors facilitate Th17 differentiation in response to cytokines like IL-6 and IL-21, the data suggest that PFS may alleviate hepatic inflammation by suppressing Th17 activation [43].

Furthermore, PFS supplementation downregulated critical hepatic genes such as *TLR4, NLRP3*, and *ATF3*, reducing activation of inflammatory pathways like NF-κB signaling and Th17 cell differentiation. TLR4 triggers inflammatory cascades via NF-κB, activating NLRP3 inflammasomes and subsequent cytokine release [44–47]. ATF3 is induced by Toll-like receptor (TLR) stimulation and functions as part of a negative feedback mechanism to limit the excessive production of proinflammatory cytokines, such as TNF [48]. Our mediation analysis demonstrated that the *NLRP3* and *ATF3* mediate the production of TNF-α with the inhibited potentially harmful species ileal bacteria such as *Bacillus cereus* and *Clostridioides*, respectively. The reduced abundance of *Bacillus cereus* and *Clostridioides* in the ileum further supports the beneficial effects of PFS on gut microbiota composition and overall animal health [49, 50]. The observed anti-inflammatory effects may, in part, be related to bioactive compounds in PFS, including luteolin and α-linolenic acid, which have been reported to influence TLR4/NF-κB signaling pathways [2, 51]. In addition to microbiota-mediated effects, the high content of ALA—an omega-3 polyunsaturated fatty acid—in PFS may independently contribute to the enhanced immune and antioxidant responses observed in our study. Recent studies demonstrate that n-3 PUFAs can directly modulate immune cell activation, reduce pro-inflammatory cytokine release, and enhance antioxidant defense through both transcriptional and post-transcriptional mechanisms, independent of microbial modulation [10, 12]. Concurrently, beneficial hepatic metabolites including chlorquinaldol, indole-3-carboxaldehyde, and swainsonine increased, contributing to antimicrobial, anti-inflammatory, and anticancer activities [52–56]. Additionally, the reductions of LysoPC(20:4) in liver and muscle by PFS feeding further underscore PFS's integrative regulatory role [57–59]. Metabolomic analysis of muscle tissue revealed a metabolic module predominantly composed of lipid metabolites (LPCs), suggesting that muscle lipid metabolism contributes to systemic immune and antioxidant modulation [60]. These findings suggest that PFS holds promise as a dietary strategy to influence gut microbiota composition and hepatic immune-metabolic pathways. Future studies should explore precise molecular mechanisms linking specific metabolites and genes to immune and antioxidant functions.

## Conclusions

Supplementation with PFS significantly enhances immune function and antioxidant capacity, evidenced by changed serum levels of immune markers (IL-10, IgM, IgG, IL-1β, TNF-α) and antioxidants (GSH-PX, SOD, MDA). Specifically, PFS promotes the proliferation of beneficial ruminal and intestinal microbes, such as *Lachnospiraceae* and *Christensenellaceae*, while concurrently suppressing potentially harmful bacteria including *Bacillus cereus* and *Clostridioides*. Moreover, liver transcriptomic and metabolomic analyses revealed that PFS downregulates critical inflammatory signaling pathways such as Fc gamma R-mediated phagocytosis and NF-κB signaling pathway mediated by TLR4, NLRP3, ATF3, NF-κB, and Th17 cells, and reduces pro-inflammatory lipid metabolites, such as LysoPC(20:4) and phosphatidic acid. These findings suggests that PFS may exert systemic immunomodulatory and antioxidant effects through the gut-liver-muscle axis, presenting a viable natural dietary intervention to sustainably enhance lamb health and productivity.

## Supplementary Information

Additional file 1: Supplementary Fig. S1. Rumen bacterial community diversity and temporal dynamics. A Dilution curves, alpha diversity indices, and beta diversity; n.s.: not significant. B–C Temporal relative abundance dynamics of dominant rumen bacterial phylaand genera.

Additional file 2: Supplementary Fig. S2. Ileal bacterial community diversity and temporal dynamics. A Dilution curves, alpha diversity indices, and beta diversity (PCoA based on weighted UniFrac distances); n.s.: not significant. B–C Temporal relative abundance dynamics of dominant ileal bacterial phyla (B) and genera (C).

Additional file 3: Supplementary Fig. S3. WGCNA linking muscle metabolites with serum health indicators. A Analysis of 86 differential muscle metabolites associated with serum indicators across LC and PFS groups. B Identification of the turquoise metabolic module highlighting lipid metabolites (phosphatidylcholines (PCs) and lysophosphatidylcholines (LPCs)) central to regulatory interactions. C The integrative multi-omics correlation analysis using regularized generalized canonical correlation analysis (RGCCA). ^*^P < 0.05, ^**^P < 0.01.

Additional file 4: Supplementary Table S1. Ingredients and nutrient composition of the LC basal diet. Supplementary Table S2. Differential expressed hepatic genes between LC and PFS groups. Supplementary Table S3. Differential hepatic metabolites between LC and PFS groups. Supplementary Table S4. Traceability of differential hepatic metabolites between LC and PFS groups based on MetOrigin.

## Data Availability

The rumen and ileum 16S rRNA raw data from this study were uploaded to the NCBI Sequence Read Archive (SRA) under the accession number PRJNA1156902 and PRJNA1156904, respectively. The raw reads of the transcriptome sequencing of the liver are available at NCBI SRA (accession number PRJNA1158691).

## Abbreviations

ALA: α-Linolenic acid
Alanine-CA: Alanine-conjugated cholic acid
ANCOM-BC: Analysis of compositions of microbiomes with bias correction
ASVs: Amplicon sequence variants
DEGs: Differentially expressed genes
DM: Dry matter
FC: Fold change
FcγR: Fc gamma receptor
FDR: False discovery rate
GCDCA: Glycochenodeoxycholic acid
GDCA: Glycodeoxycholic acid
GHDCA: Glycohyodeoxycholic acid
GLU: Glucose
GSEA: Gene set enrichment analysis
GSH-Px: Glutathione peroxidase
GUDCA: Glycoursodeoxycholic acid
IDA: Information-dependent acquisition
IgA: Immunoglobulin A
IgG: Immunoglobulin G
IgM: Immunoglobulin M
IL-10: Interleukin-10
IL-1β: Interleukin-1β
KEGG: Kyoto Encyclopedia of Genes and Genomes
LC: Low-concentrate control diet
LEfSe: Linear discriminant analysis effect size
LPCs: Lysophosphatidylcholines
LPE: Lysophosphatidylethanolamine
MDA: Malondialdehyde
n-3 PUFA: Omega-3 polyunsaturated fatty acid
NH_3_-N: Ammonia-nitrogen
OPLS-DA: Orthogonal projections to latent structures-discriminant analysis
PCoA: Principal Coordinates Analysis
PCs: Phosphatidylcholines
PFS: *Perilla frutescens *seeds
QC: Quality control
RGCCA: Regularized generalized canonical correlation analysis
SOD: Superoxide dismutase
SYK: Spleen tyrosine kinase
TG: Triglyceride
TGF-β: Transforming growth factor-β
TLR: Toll-like receptor
TNF-α: Tumor necrosis factor-alpha
VFAs: Volatile fatty acids
VIP: Variable importance in projection
WGCNA: Weighted gene co-expression network analysis
