# Supplementary figures and images for: Perilla frutescens seeds enhance lamb immunity and antioxidant capacity via the microbiota-gut-liver-muscle axis
Source: J Anim Sci Biotechnol. 2026 Jan 4;17:1. doi: 10.1186/s40104-025-01317-3 (PMC12765310; doi:10.1186/s40104-025-01317-3)

A

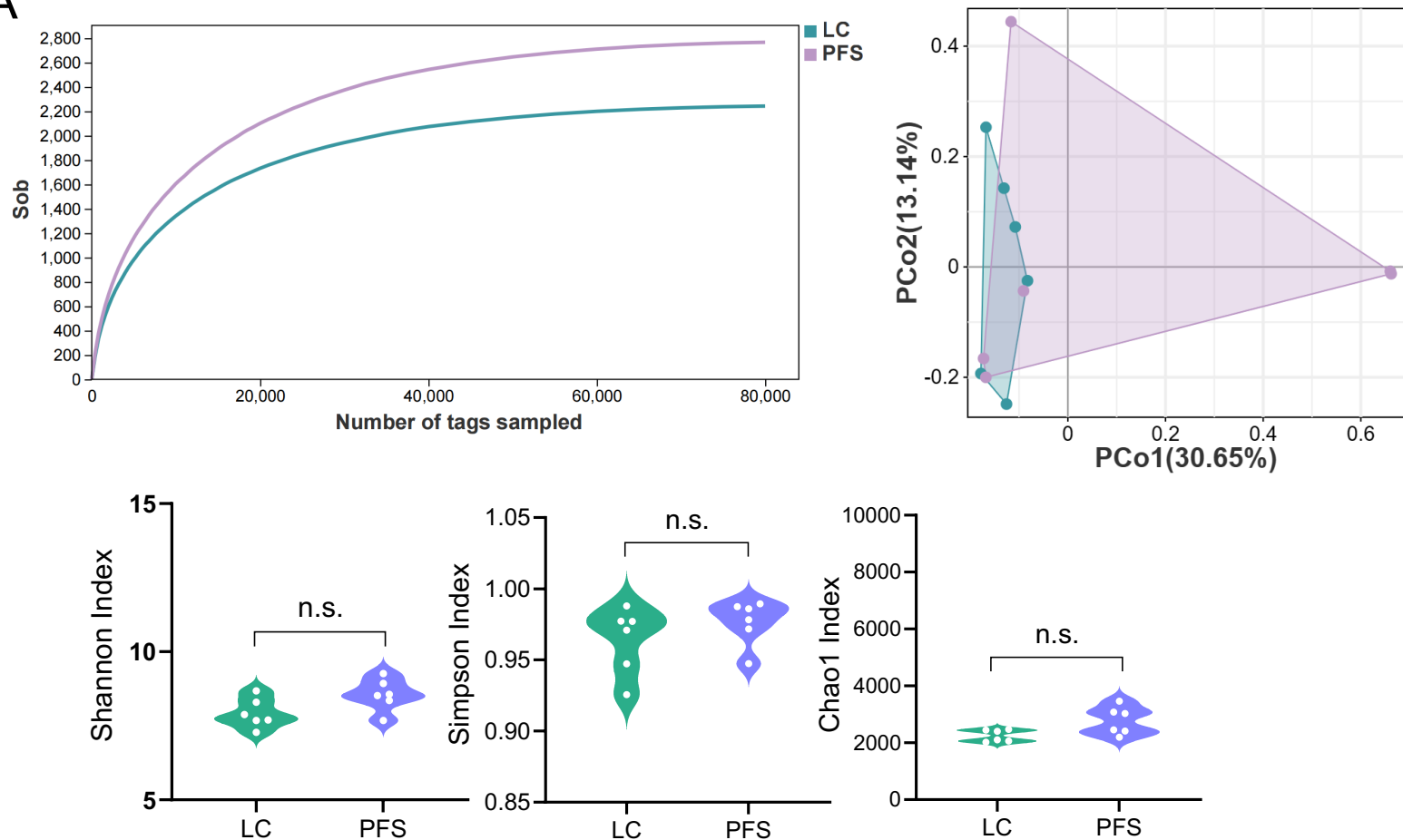

B

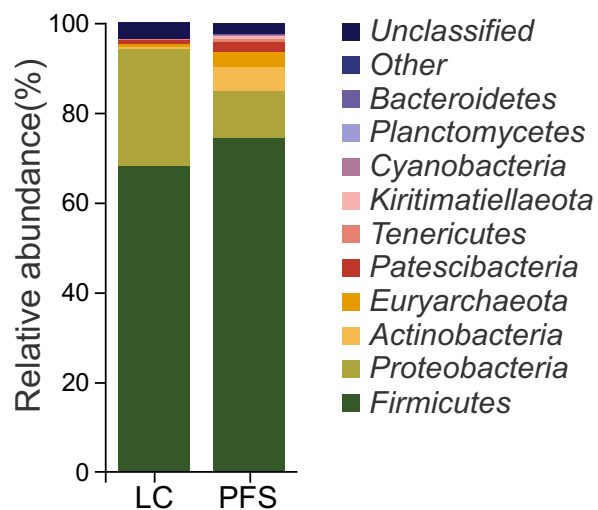

C

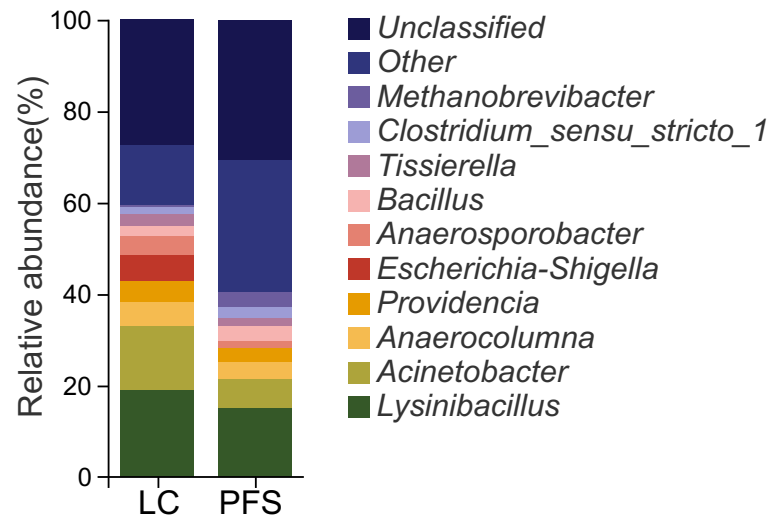

Supplement: Supplementary file 2 — Additional file 2: Supplementary Fig. S2. Ileal bacterial community diversity and temporal dynamics. A Dilution curves, alpha diversity indices, and beta diversity (PCoA based on weighted UniFrac distances); n.s.: not significant. B–C Temporal relative abundance dynamics of dominant ileal bacterial phyla (B) and genera (C). [file 40104_2025_1317_MOESM2_ESM.pdf]

A

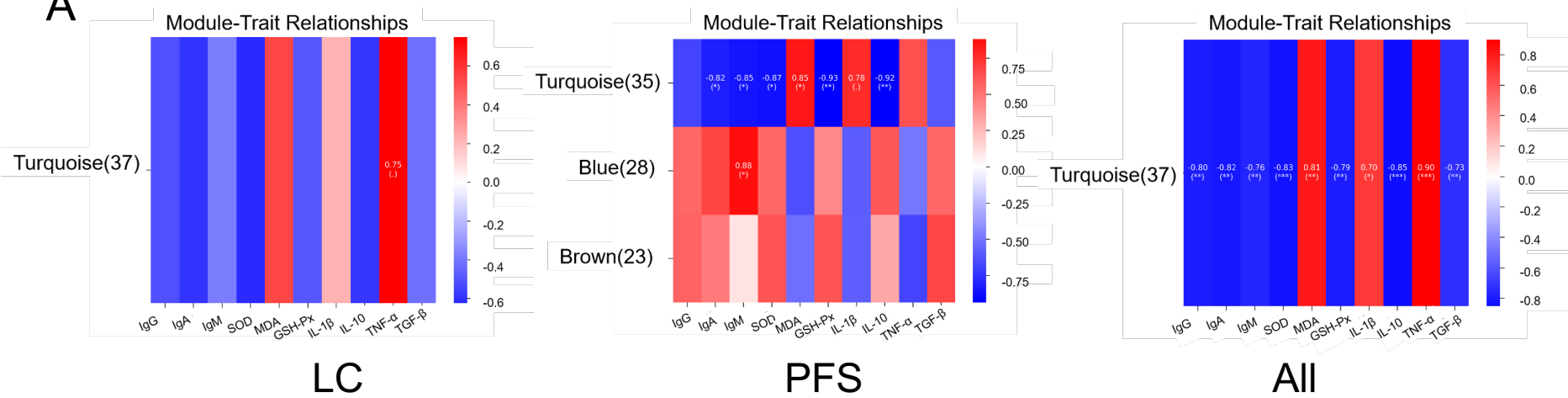

B

## Core turquoise module

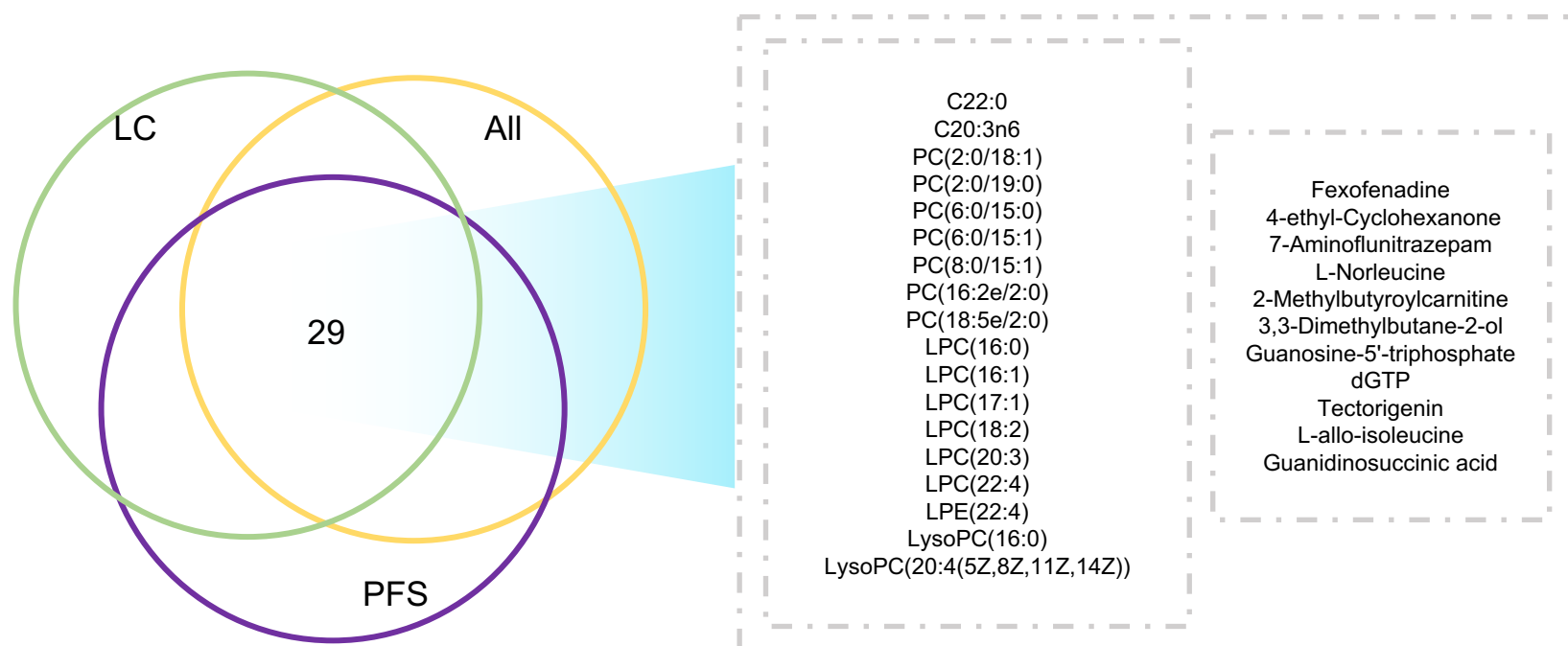

C

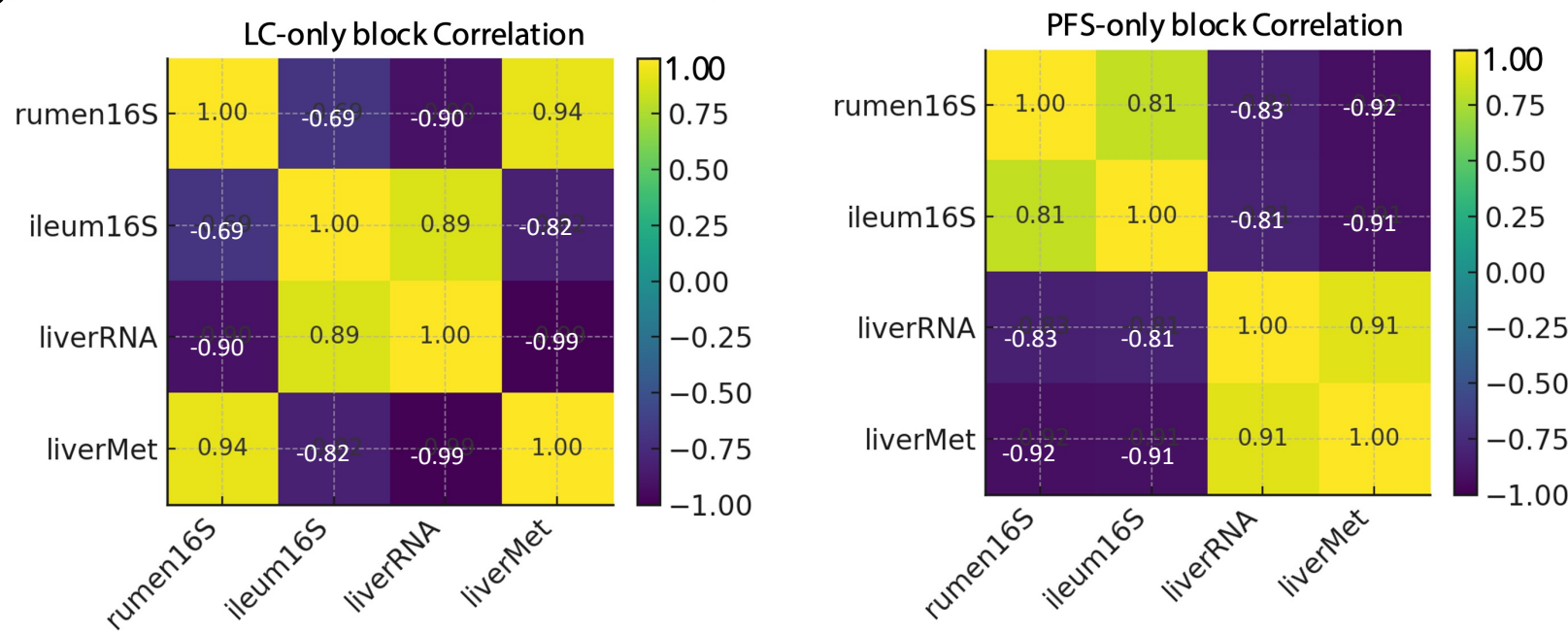

Supplement: Supplementary file 3 — Additional file 3: Supplementary Fig. S3. WGCNA linking muscle metabolites with serum health indicators. A Analysis of 86 differential muscle metabolites associated with serum indicators across LC and PFS groups. B Identification of the turquoise metabolic module highlighting lipid metabolites (phosphatidylcholines (PCs) and lysophosphatidylcholines (LPCs)) central to regulatory interactions. C The integrative multi-omics correlation analysis using regularized generalized canonical correlation analysis (RGCCA). *P < 0.05, **P < 0.01. [file 40104_2025_1317_MOESM3_ESM.pdf]
